# Cyclic Neutropenia Mimicking Crohn’s Disease: Two Case Reports and a Narrative Review

**DOI:** 10.3390/jcm12196323

**Published:** 2023-09-30

**Authors:** Alessia Dalila Guarino, Gaetano Luglio, Nicola Imperatore, Giuseppe Cerciello, Novella Pugliese, Fabiana Castiglione, Francesca Paola Tropeano, Anna Testa, Oriana Olmo, Antonio Rispo

**Affiliations:** 1 Gastroenterology, Department of Clinical Medicine and Surgery, “Federico II” University, 80131 Naples, Italy; ale.tizi@hotmail.it (A.D.G.);; 2 Endoscopic Surgery, Department of Clinical Medicine and Surgery, “Federico II” University, 80131 Naples, Italy; 3 Gastroenterology,“Santa Maria Delle Grazie” Hospital, 80078 Pozzuoli, Italy; 4 Hematology, “San Pio” Hospital of Benevento, 82100 Benevento, Italy; 5 Hematology, Department of Clinical Medicine and Surgery, “Federico II” University, 80131 Naples, Italy

**Keywords:** Crohn’s disease, IBD, cyclic neutropenia, leukopenia, surgery

## Abstract

Cyclic neutropenia is a rare hematological condition characterized by periodic fluctuations in neutrophil counts, with a 21-day periodicity. Clinical presentation varies from mild to severe forms of the disease, with the onset of recurrent fever, painful oral ulcers, recurrent bacterial infections, peritonitis, and septic shock. The availability of granulocyte colony-stimulating factor (G-CSF) has revolutionized the management and natural history of this disease, regulating the proliferation, differentiation, and maturation of the progenitor cells, and reducing the duration of neutropenia. Inflammatory bowel disease (IBD), including Crohn’s disease (CD) and ulcerative colitis (UC), is a group of chronic pathologies that affect the gastrointestinal tract. The onset of both diseases may be at a young age (even during childhood or adolescence), and clinical manifestations may lead to misdiagnosis, due to similar characteristics such as recurrent infections, oral ulcers, perianal abscesses, and infertility. Moreover, the two pathologies are rarely associated, with different management and therapeutic options. Here, we describe two case reports of patients who underwent surgery because of diagnosis of complicated CD. After surgery, due to persistent neutropenia, the hematologist consultant confirmed suspicions of cyclic neutropenia, and G-CSF therapy was started with benefits, underlining the crucial importance of proper differential diagnosis.

## 1. Background

Cyclic neutropenia is a rare hematological disorder characterized by periodic fluctuations in blood neutrophil counts, with a 21-day turnover frequency [1,2,3].

Neutrophils are white blood cells (WBC) involved in immune defense, playing a crucial role in ingestion and killing of microorganisms and destruction of intra-cellular pathogens and the alteration of their functions determines immunodeficiency with consequent development of recurrent infections [1,4,5,6].

The genetic basis of the disease has been evaluated and established in molecular biology, showing that cyclic neutropenia is inherited as an autosomal-dominant mutation of the gene for neutrophil elastase (ELANE) with full penetrance but different severities of manifestation [1,2,4,7].

Clinical presentation ranges from mild to severe forms of the disease, with an absolute neutrophil count <0.2 × 10^9^/L for a period of 3–5 days, recurrent fever, painful mouth ulcers, gingivitis, and bacterial infections such as sinusitis, otitis, pharyngitis, and cellulitis [1,3,8]. Young patients can also manifest periodontitis and alveolar bone loss; pneumonia, perianal abscesses, impaired fertility, septic shock, and bone necrosis represent rare manifestations of the disease; symptoms usually become less severe and are less frequent after adolescence [1,2,3,9].

Effective and safe therapy with granulocyte colony-stimulating factor (G-CSF) has revolutionized the management of the disease, consisting of daily or alternate daily subcutaneous injection, which can reduce the neutropenia duration and cell turnover [1,2,6,8].

Crohn’s disease (CD) is a chronic inflammatory bowel disease (IBD) that affects the gastrointestinal tract, with an incidence increasing worldwide [10,11,12]. Symptoms include diarrhea, abdominal pain, fever, weight loss, and malnutrition; extraintestinal manifestations (EIMs) are frequently reported, affecting up to 40% of patients [10,13,14].

Endoscopy and imaging techniques, such as bowel ultrasound, small bowel magnetic resonance imaging (MRI), and computed tomographic (CT) enterography, are the tools for CD’s diagnosis and management [10]. The focus of IBD treatment has changed in recent years, with the introduction of biological therapies, moving from the treatment of symptoms to “deep remission” [10,11,15,16,17].

These chronic diseases affect young people and—due to their relapsing/remitting course, the presence of EIMs, and the necessity of surgery—have an important impact on the quality of life and the development of disabilities [16,18].

The association between CD and cyclic neutropenia is rarely reported in the literature; differential diagnosis may be very complex, since they are both chronic diseases with non-specific, often overlapping symptoms that affect young people.

Here, we describe the clinical cases of two young men diagnosed with CD, who underwent surgery because of complicated disease. After surgery, they were diagnosed with cyclic neutropenia, and they both started G-CSF, with normalization of blood cell counts and improvement of their gastrointestinal symptoms.

## 2. Case Report 1

In 2020, a 31-year-old patient referred chronic diarrhea and right low quadrant pain, associated with recurrent mouth ulcers. He mentioned that he had been suffering from recurrent otitis since childhood, but no laboratory tests or medical consultation were performed.

Ultrasound was performed, but it did not show any abnormalities in the abdominal organs. Therapy with antibiotics and probiotics was prescribed, but there was no improvement in clinical symptoms. In 2021, he came to our attention in our Gastroenterological Department with worsening abdominal pain and fever (up to 39 °C). Physical examination revealed abdominal tenderness and guarding in the right lower quadrant; bowel ultrasound was performed, showing increased bowel wall thickness of the ileum and the presence of a peri-ileal phlegmon. The patient underwent a computed tomography (CT) scan and ileo-colonoscopy, with evidence of penetrating ileal CD.

Laboratory tests gave the following results: WBC 2500 × 10^3^/UL (neutrophils 39%) and C-reactive protein (CRP): 45 mg/L; this data was interpreted to be as a consequence of a gram-negative septic process. In 2021, the patient underwent ileo-colic resection with temporary ileostomy with post-operative histology showing fibrosis, congestion, and lymphoplasmacytic inflammatory infiltrate.

**Figure 1:**
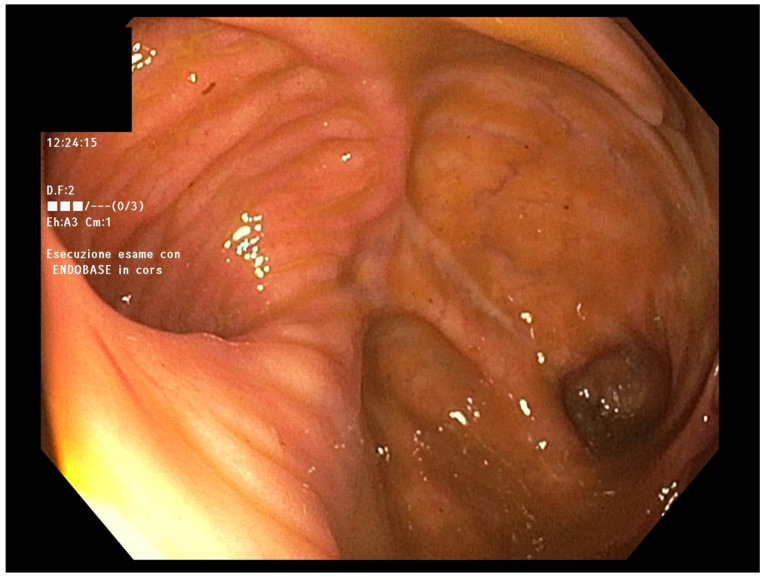
Normal anastomotic picture at endoscopy.

In April 2022, endoscopic evaluation was performed (Figure 1), without showing any abnormalities in mucosal anastomosis but mild mucosal erythema of the left colon.

On outpatient visit, he referred to the absence of abdominal pain and fever but the persistence of two to three bowel movements/day; laboratory tests showed that leukopenia was still present (WBC: 1.93 × 10^3^/UL; neutrophils: 40%), and one month later, his WBC count was found to be stable at 2.100 × 10^3^/UL (neutrophils: 37%).

The patient had not used any medications, toxins, or alcohol, and there was no family history of neutropenia.

Therefore, a hematological evaluation was required for suspected CD-like ileitis in patients with cyclic neutropenia and the hematologist at our hospital confirmed the diagnosis. The previous series of patient’s laboratory tests were evaluated, which clearly showed the presence of cyclic neutropenia, with a 21-day turnover frequency (Table 1), but additional tests, such as the detention of ELANE mutation, were not performed. G-CSF was started, leading to normalization of blood cell counts and alleviation of intestinal symptoms.

In November 2022, he underwent surgery for ileostomy reversal; laboratory tests were in range; in particular, blood routine tests gave a WBC count of 7.33 × 10^3^/UL.

At the time of writing, the patient is well and still undergoing therapy with G-CSF, without any recurrence of neutropenia or endoscopic and clinical signs of ileitis or Crohn’s disease.

## 3. Case Report 2

In 2017, a 25-year-old patient with a history of recurrent pharyngitis and oral aphthos is since adolescence, presented with abdominal pain and diarrhea.

He had never performed laboratory or any other diagnostic tests, so ileo-colonoscopy with biopsies and magnetic resonance enterography (MRE) were performed, and a diagnosis of ileo-colic Crohn’s disease (CD) was made. He started therapy with mesalamine and a cycle of steroids, referring partial clinical benefits.

In 2019, after clinical recurrence of the disease, ileo-colonoscopy and MRE were performed again, showing the presence of stenosing ileo-colic CD. Histological examination of colic specimens confirmed active CD, but no surgical evaluation was made.

In these 24 months, laboratory tests showed persistence of neutropenia (WBC ranged from 1.900 × 10^3^ UI/L to 2.900 × 10^3^ UI/L; neutrophils ranged from 30% to 49%) (Table 2). A hematological consultant suggested a bone marrow biopsy, and the results appeared normal.

**Figure 2:**
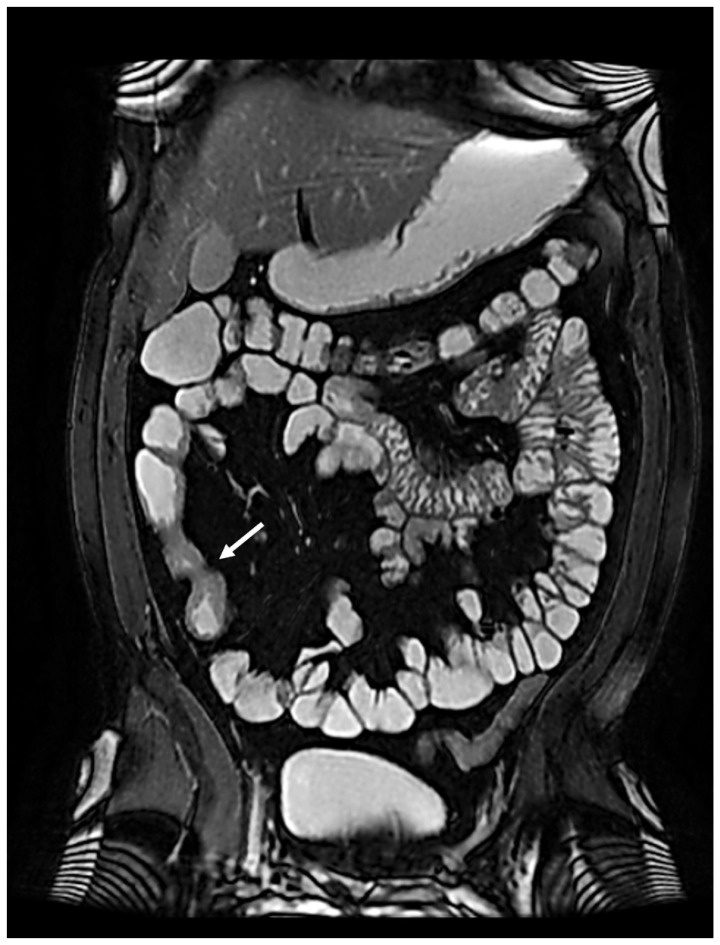
Suspected stenosing Crohn’s disease at MRE (arrow).

In January 2022, the patient complained of worsening abdominal pain localized in the right low quadrant, so ileo-colonoscopy and MRE were performed again, confirming stenosis of the terminal ileum and ascending colon (Figure 2).

In December 2022, he was reported to our Gastroenterological Department; bowel ultrasound was performed, showing bowel thickening in the ileum, caecum, and ascending colon, with the presence of an ileal-mesenteric fistula. In January 2023, he underwent ileo-colic resection at our Surgical Department and after surgery, WBC remained stable between 2.43 × 10^3^/UL and 2.85 × 10^3^/UL with associated evidence of neutropenia.

After hematological consultation with evaluation of the series of patient blood cell counts, the diagnosis changed from CD to cyclic neutropenia, and G-CSF therapy was started with rapid normalization of WBC. At the time of writing this report (6 months later), his condition is still stable, with no intestinal symptoms. No other molecular tests were performed and no endoscopic sign of inflammation is evident at the site of ileo-colic anastomosis.

## 4. Narrative Review and Discussion

Neutrophil cells are the most represented sub-population of leukocytes, constituting the first line of the innate immune system, and implicated in the release of inflammatory chemical mediators such as leukotrienes, prostaglandins, proteases, and free radicals [1,4,5,6]. They can ingest and kill microorganisms, destroy intracellular pathogens, and degrade proteins such as immunoglobulins and coagulation factors [1,4,5,6].

In the general population, the blood neutrophil count ranges from 2.0 × 10^9^/L to 7.0 × 10^9^/L, deriving from the hematopoietic stem cells (HSC) that differentiate into myeloid progenitor cells, which differentiate into granulocyte–monocyte, and then into neutrophils [2,3]. Granulocyte colony-stimulating factor (G-CSF) represents the main factor involved in the proliferation and maturation of neutrophils in the bone marrow [3].

Neutropenia is a condition defined as a decrease in the neutrophil count below 2.0 × 10^9^/L, while severe neutropenia is characterized by neutrophil counts less than 0.5 × 10^9^/L, leading to elevated susceptibility to infections [1,2].

Cyclic neutropenia is a rare, benign hematological condition, affecting one in a million people in the general population [1,2,3]. It is characterized by periodic fluctuations in neutrophil counts, with a 21-day periodicity; alteration of the neutrophil level leads to immunodeficiency and consequent development of recurrent infections [1,2,3].

In recent years, research in molecular biology has led to an understanding of the genetic basis of the disease, which is inherited as an autosomal-dominant mutation of the gene for neutrophil elastase (ELANE), with full penetrance but different severities of manifestations [1,2,4]. Elastase is one of the four serine proteases found in neutrophil granules, and its defect determines the cessation of myelocyte maturation [3,4]. The mutation can be found in all familiar members affected by cyclic neutropenia, with no differences according to sex, and it is not detectable in unaffected familiar members [3,4].

Linkage analysis has shown that the alteration of the ELANE gene (also referred to as ELA2, HLE, or NE) is localized in the chromosome 19p13.3, in the exon 4 or 5 of the gene, or at the junction of exon 4 and intron 4, determining aberrant function with alteration in the development of the myeloid precursor, as well as cell loss and neutropenia [1,3,4,8].

Heterozygous mutation of the gene determines the onset of cyclic neutropenia, with a mild–moderate form of the disease, as well as severe neutropenia, which is life-threatening [3]; sporadic cases can occur in patients with so-called acquired neutropenia, with late onset of the disease [2].

The pathophysiological mechanism is attributable to an intrinsic defect in the hematopoietic stem cells, leading to cyclic oscillation in the maturation of neutrophils, with a pause at the promyelocyte or myelocyte stage [3]; according to a mathematical model proposed by Mackey, oscillation in count cells is the consequence of impaired hematopoiesis in acquired neutrophilia [1,19].

Clinical presentation can vary from mild to severe form of the disease, including recurrent severe neutropenia with evidence of absolute neutrophil count <0.2 × 10^9^/L for 3–5 days, recurrent fever, oral mucosal ulcers, gingivitis, and recurrent bacterial infections such as sinusitis, otitis, pharyngitis, and cellulitis [1,3,8].

Opportunistic infections may also occur as dermatological infections, and young patients can manifest swollen lymph nodes, abdominal pain, digestive system infections, fatigue, periodontitis, and alveolar bone loss [2]. Pneumonia, perianal abscesses, and impaired fertility affecting women but not men are rarely present [2]; women show a higher rate of abortions and 50% of their children inherit cyclic neutropenia [2,3]. Septic shock, peritonitis, and bone necrosis represent the more serious but rare manifestations of the disease [2,3,9]. Symptoms usually become less severe and less frequent after adolescence and therapy with antibiotics is frequently required, but cyclic neutropenia is not associated with increased risk of malignancy or conversion to leukemia [2,3].

Diagnosis in children may be a challenge for physicians, due to the non-specificity of symptoms and may require a multidisciplinary approach involving a hematologist, a gastroenterologist, an infectious disease specialist, a geneticist, a radiologist, and a dentist [4].

A proper diagnostic flowchart includes the evaluation of family history, clinical symptoms and oncological markers, blood counts, bone biopsy, X-ray, ultrasound, and computerized tomography (CT) [4]; moreover, a DNA study may be used to confirm the diagnostic suspicion [4].

Regarding laboratory tests, blood counts should be obtained 3 days a week for 6 weeks, with demonstration of counts below 0.2 × 10^9^/L [2].

A diagnostic dilemma for the physician is represented by differential diagnoses of several conditions, such as infectious disorders, juvenile idiopathic arthritis, Bechet’s disease, Mediterranean fever syndrome, and other hereditary disorders, but also using drugs, as well as chemotherapy [2,4].

Different therapeutic approaches were tried in the past, including splenectomy, androgens, steroids and lithium, without any success [6], but the availability of granulocyte colony-stimulating factor (G-CSF) has revolutionized the management and natural history of cyclic neutropenia [8]; administration of myeloid growth factor or G-CSF is currently the most efficacious therapy for cyclic neutropenia, with a good clinical response [8]. It consists of daily or alternate daily subcutaneous injection and can regulate the proliferation, differentiation, and maturation of the progenitor cells, reducing the duration of neutropenia and shortening the turnover of the cells form 21 days to 14 days [2,6], with the usual dose of 2–5 mg/kg/day, which is well tolerated by patients [2,6]. Adverse events are dose-dependent and commonly represented by headaches, mild bone pain, and osteoporosis, but the development of myeloid leukemia is not considered a complication of the disease [2,6].

Granulocyte–macrophage colony-stimulating factor (GM-CSF) has been used as therapy for cyclic neutropenia, but it seems to have less efficacy than G-CSF [1].

Crohn’s disease (CD) is a transmural chronic inflammatory bowel disease (IBD) that affects the gastrointestinal tract, especially the terminal ileum and colon [11,12]. Clinical manifestations including diarrhea, abdominal pain, fever, weight loss, anorexia, and extraintestinal manifestations (EIMs) are also frequently reported, affecting up to 40% of patients [10]. The etiopathogenesis of this IBD is still unclear, but the association with genetic and environmental factors has been demonstrated [20,21,22]; the incidence is increasing worldwide, with about 2.5 million people affected in Europe, especially young adults [23,24,25].

CD diagnosis is often challenging and requires the definition of location, extent, severity, and type of disease, as well as the exclusion of complications and EIMs [10,11,14]. Endoscopy, imaging techniques (bowel ultrasound, small bowel MR, or CT enterography) and non-invasive disease markers (for example: fecal calprotectin) are tools used for the diagnosis and evaluation of the remission/recurrence of the disease [10,14]. Since the advent of targeted biologic therapies, the therapeutic landscape of IBD has undergone a radical transformation, with the goal of inducing and maintaining remission [11]. A better understanding of the mucosal immune system and the genetics involved in the pathogenesis of IBD in recent decades has led to a more ambitious disease control strategy that can change the natural course of the pathology [11].

The complexity of CD, the chronic relapsing course, the young age of most patients at diagnosis, the variability of therapeutic effects, the risk of adverse events, and the frequent need for surgery greatly impact a patient’s quality of life and tendency to develop a disability [16,18]. According to one intriguing theory, judging by the impaired inflammatory response and altered role of macrophages in the removal of pathogens, IBD may be considered as a stage of a complex immunodeficiency [20,22]. Rare cases of early onset IBD are associated with a phagocyte immunodeficiency background, and some genes related to phagocyte function are linked to IBD [20,22]. The development of an irregular inflammatory response has led to the consideration of IBD as a model disease in order to recognize factors that regulate the mucosal immune system [20,22].

The association between CD and cyclic neutropenia is a very rare condition; Fata et al. described a case of a 40-year-old patient who, in 1985, was diagnosed with ileo-colic CD associated with cyclic neutropenia, but not with any immunosuppressive therapy [6]. Routine blood tests from 1989 revealed the presence of neutropenia but bone marrow aspirations and biopsies, at two different times, showed no form of alteration [6]. In 1995, the patient had central catheter (used for parenteral nutrition) infection caused by *Staphylococcus aureus*, which was treated by intravenous administration of vancomycin for 4 weeks and by removal of the catheter [6]. Evidence of persistence of cyclic neutropenia with a 14-day turnover, despite CD remission, led to the adoption of G-CSF therapy, with resolution of the sepsis [6]. An attempt to interrupt G-CSF therapy led again to reduced neutrophil counts, but the neutrophils increased in range when G-CSF was restarted [6].

Lamport described another case of a woman affected by CD associated with neutropenia whose diagnosis became clear only when CD was in remission and the patient had stopped any therapy [26].

Dale et al. reported a case of a 10-year-old boy who had recurrent infections (otitis, pneumonia, and pharyngitis) in early childhood and was diagnosed with cyclic neutropenia [27]. Lithium therapy was started and four months later, he complained of abdominal pain and chills [27]. He was hospitalized and diagnosed with acute abdomen with septic shock complication [27]. Urgent colectomy was performed, but unfortunately the child died few hours later; autopsy revealed a necrotizing enterocolitis with *Clostridium perfringens* infection [27].

These cases in the literature show how cyclic neutropenia may manifest not only with mild recurrent infections but also with severe clinical conditions, such as sepsis or fatal enterocolitis, underlining the crucial role of proper diagnosis and treatment [27]. An important point to focus on is the differential diagnosis of cyclic neutropenia and CD, since they share similar symptoms/signs and similar alteration in the laboratory tests. In fact, neutropenia can be common laboratory evidence in CD, because of infection and some drugs; similarly, neutropenia may show some clinical features common in CD, such as recurrent fever, oral mucosal ulcers, or perianal disease [1,2,3,9].

Septic shock due to severe infections represents rare complications of both diseases and digestive system infections, abdominal pain, and fatigue are common findings in CD and cyclic neutropenia [2,3,8,9]. Other clinical features such as periodontitis, alveolar bone loss, and cellulitis are characteristics of cyclic neutropenia but not of CD [2,3,8,9]. The overlap of the features may determine the occurrence of misdiagnosis as in our two clinical cases, so specialists should always be aware of the possibility of differential diagnosis [1,8,9,26].

In cyclic neutropenia, endoscopy shows the presence of hyperemia, oedema, erosions, and ulcers in the colonic mucosa; histology shows severe inflammation with lympho-plasmocytic/eosinophilic infiltrate and the absence of neutrophils in the lamina propria and in the glands [4,6,9,19,27].

In our two case reports, histology was at first suggestive of CD, but in effect many histological signs of intestinal inflammation are not specific to IBD, and can be present even in other chronic intestinal diseases; therefore, a critical revision of the clinical cases and the patients’ clinical features supported the change of diagnosis to cyclic neutropenia.

Gastroenterologists should be careful when a patient with CD shows laboratory evidence of neutropenia, particularly if it is present during the remission phases of intestinal disease, and the patient does not take drugs capable of inducing neutropenia (e.g., thiopurines). Moreover, particular attention should be paid when, for any other apparent reasons, the neutrophil counts have a periodicity with a turnover of 21 days.

## 5. Conclusions

CD and cyclic neutropenia are two chronic diseases with a heterogeneous, often overlapping spectrum of symptoms that affect young adults. Their differential diagnosis is difficult and only a few cases of association between CD and neutropenia are described in the literature. Nevertheless, the existence of conditions of undiagnosed cyclic neutropenia mimicking CD, as in our experience, is plausible. In this regard, a correct anamnestic collection aimed at assessing the presence of recurrent and periodic infections, the evaluation of laboratory tests and instrumental examinations, and possible genetic analysis may lead to a proper diagnosis and correct therapeutic approach.

## Figures and Tables

**Table 1:** White blood cells showing the presence of 21-day-turnover frequency (case report 1).

Date	White Blood Cells (Neutrophils)
9 May 2022	1.93 × 10^3^/UL (40%)
11 May 2022	1.76 × 10^3^/UL (40%)
13 May 2022	1.87 × 10^3^/UL (40%)
16 May 2022	1.97 × 10^3^/UL (40%)
18 May 2022	1.81 × 10^3^/UL (40%)
20 May 2022	1.45 × 10^3^/UL (40%)
23 May 2022	1.74 × 10^3^/UL (40%)
25 May 2022	1.52 × 10^3^/UL (40%)
27 May 2022	1.76 × 10^3^/UL (40%)
30 May 2022	2.31 × 10^3^/UL (37%)
1 June 2022	2.53 × 10^3^/UL (34%)
3 June 2022	2.67 × 10^3^/UL (39%)
6 June 2022	3.18 × 10^3^/UL (40%)
8 June 2022	2.12 × 10^3^/UL (39%)
11 June 2022	2.19 × 10^3^/UL (41%)
13 June 2022	3.22 × 10^3^/UL (42%)
15 June 2022	2.18 × 10^3^/UL (37%)
17 June 2022	2.10 × 10^3^/UL (41%)
20 June 2022	1.98 × 10^3^/UL (38%)
22 June 2022	2.13 × 10^3^/UL (36%)
24 June 2022	2.17 × 10^3^/UL (40%)

**Table 2:** White blood cells showing the presence of 21-day-turnover frequency (case report 2).

Date	White Blood Cells (Neutrophils)
6 July 2020	1.89 × 10^3^/UL (41%)
9 July 2020	1.93 × 10^3^/UL (42%)
11 July 2020	1.97 × 10^3^/UL (46%)
13 July 2020	1.78 × 10^3^/UL (39%)
15 July 2020	1.65 × 10^3^/UL (37%)
17 July 2020	1.97 × 10^3^/UL (41%)
20 July 2020	1.86 × 10^3^/UL (36%)
22 July 2020	1.81 × 10^3^/UL (38%)
24 July 2020	1.95 × 10^3^/UL (41%)
27 July 2020	2.15 x 10^3^/UL (45%)
28 July 2020	2.45 × 10^3^/UL (47%)
31 July 2020	2.52 × 10^3^/UL (46%)
3 August 2020	2.76 × 10^3^/UL (48%)
5 August 2020	2.73 × 10^3^/UL (45%)
7 August 2020	2.68 × 10^3^/UL (41%)
10 August 2020	2.81 × 10^3^/UL (48%)
12 August 2020	2.90 × 10^3^/UL (43%)
14 August 2020	2.69 × 10^3^/UL (40%)
17 August 2020	1.91 × 10^3^/UL (37%)
19 August 2020	1.83 × 10^3^/UL (39%)
21 August 2020	1.75 × 10^3^/UL (40%)
